# Huge osteoma in the frontoparietal bone caused by trauma from 18 years ago: a case report

**DOI:** 10.1186/s13256-024-04373-x

**Published:** 2024-02-09

**Authors:** Yamama Tawashi, Kenana Tawashi, Osama Ahmad, Aref Hamada, Khaleel Alhakeem

**Affiliations:** 1 Faculty of Medicine, Hama University, Hama, Syria; 2 Neurosurgery Department, Hama National Hospital, Hama, Syria

**Keywords:** Huge osteoma, Frontal bone, Parietal bone, Trauma, Case report

## Abstract

**Background:**

Osteomas are asymptomatic, benign tumors and are diagnosed accidentally by radiological investigations conducted for other reasons. In some cases, they may cause aesthetic or functional symptoms by affecting nearby organs. The cause of osteoma is still dialectical. Many theories suggest that inflammation, trauma, or congenital causes are behind its formation. In our case, the patient presented with a symptomatic and huge osteoma in the frontoparietal bone caused by trauma from 18 years ago.

**Case presentation:**

A 24-year-old Syrian woman came to our hospital complaining of headaches, syncope episodes, blurred vision, and tumor formation in the frontoparietal region. The medical and surgical histories of the patient revealed appendectomy and head trauma when she was 6 years old in a traffic accident. Radiological investigations showed thickness in the space between the two bone plates in the left frontoparietal region, which reached the orbital roof without cortical destruction or periosteum reaction; the tumor size was 5 cm × 5 cm. A surgical excision was indicated. Under general anesthesia, the surgery was done for the tumor excision. The histopathology examination emphasized the diagnosis of osteoma. The follow-up for 7 months was uneventful.

**Conclusion:**

This paper highlights the importance of focusing on the medical history of patients with osteoma in an attempt to explain the reasons for its occurrence. It stresses the need to put osteoma within the differential diagnoses of skull tumors.

## Background

Osteomas are benign tumors that contain both cortical and cancellous bone tissues [1, 2]. They are the most common benign tumors in the paranasal sinuses; the most affected one is the frontal sinus, followed by ethmoid, maxillary, and sphenoid sinuses [3]. Cranial osteomas are classified into the skull base, skull vault, dural (arising from the falx), and intraparenchymal (without any connection to dura or bone) [4]. They are also classified depending on their site into central (deriving from endosteum), peripheral (deriving from periosteum), and extraskeletal (developing inside soft tissues) [5]. Osteomas are usually asymptomatic, and they are diagnosed accidentally by radiological investigations conducted for other reasons. In some cases, they may cause aesthetic or functional symptoms by affecting nearby organs [1]. The cause of osteoma is still dialectical. Many theories suggest inflammation, trauma, or congenital causes are behind its formation [4, 6, 7]. In our case, the patient presented with a huge osteoma in the frontoparietal bone caused by trauma from 18 years ago.

## Case presentation

**Fig. 1:**
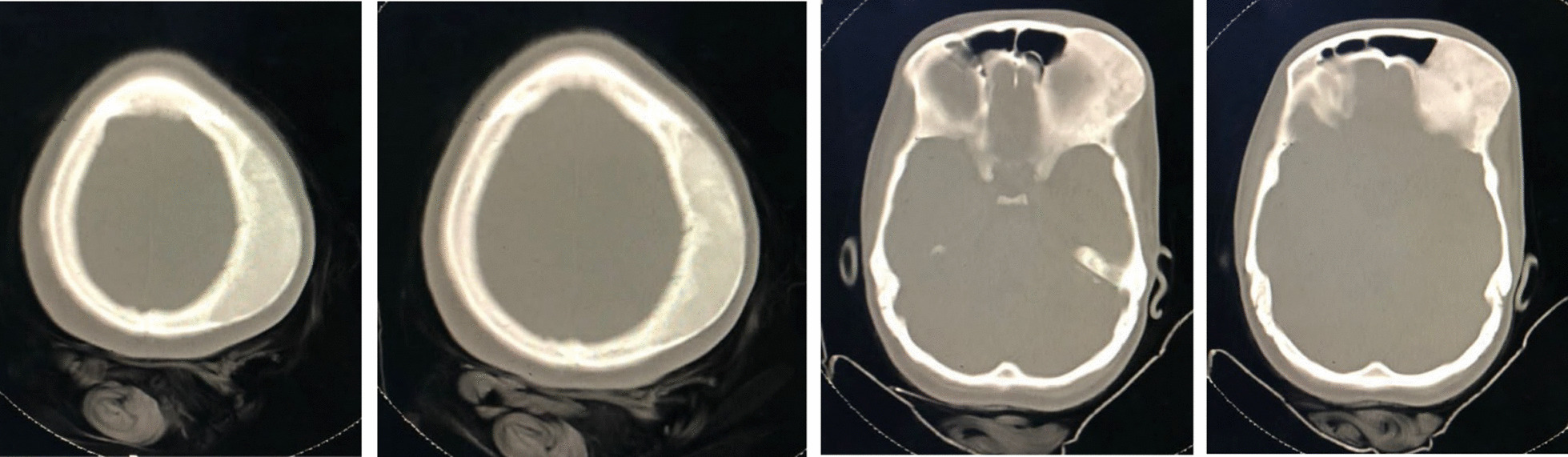
Head CT scan showing thickness in the space between the two bone plates in the left frontoparietal region, which reached the orbital roof without cortical destruction or periosteum reaction; the tumor size was 5 cm × 5 cm

**Fig. 2:**
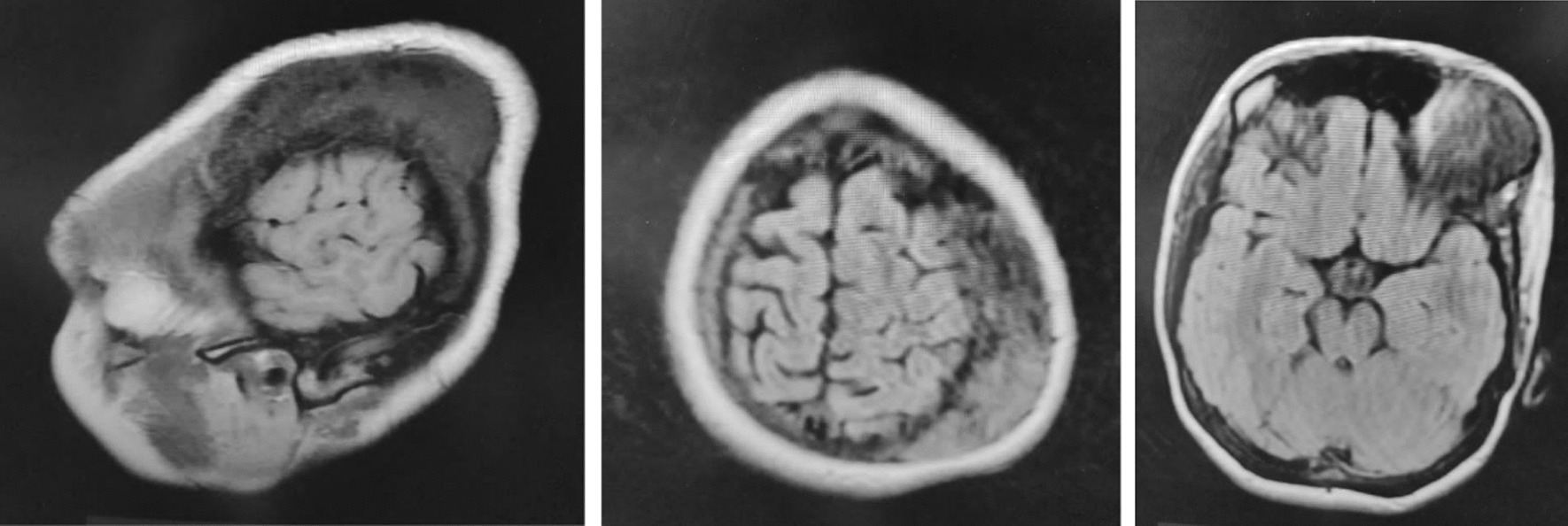
MRI showing the heterogeneous bony tumor in the left frontoparietal region; the brain was within normal parameters

**Fig. 3:**
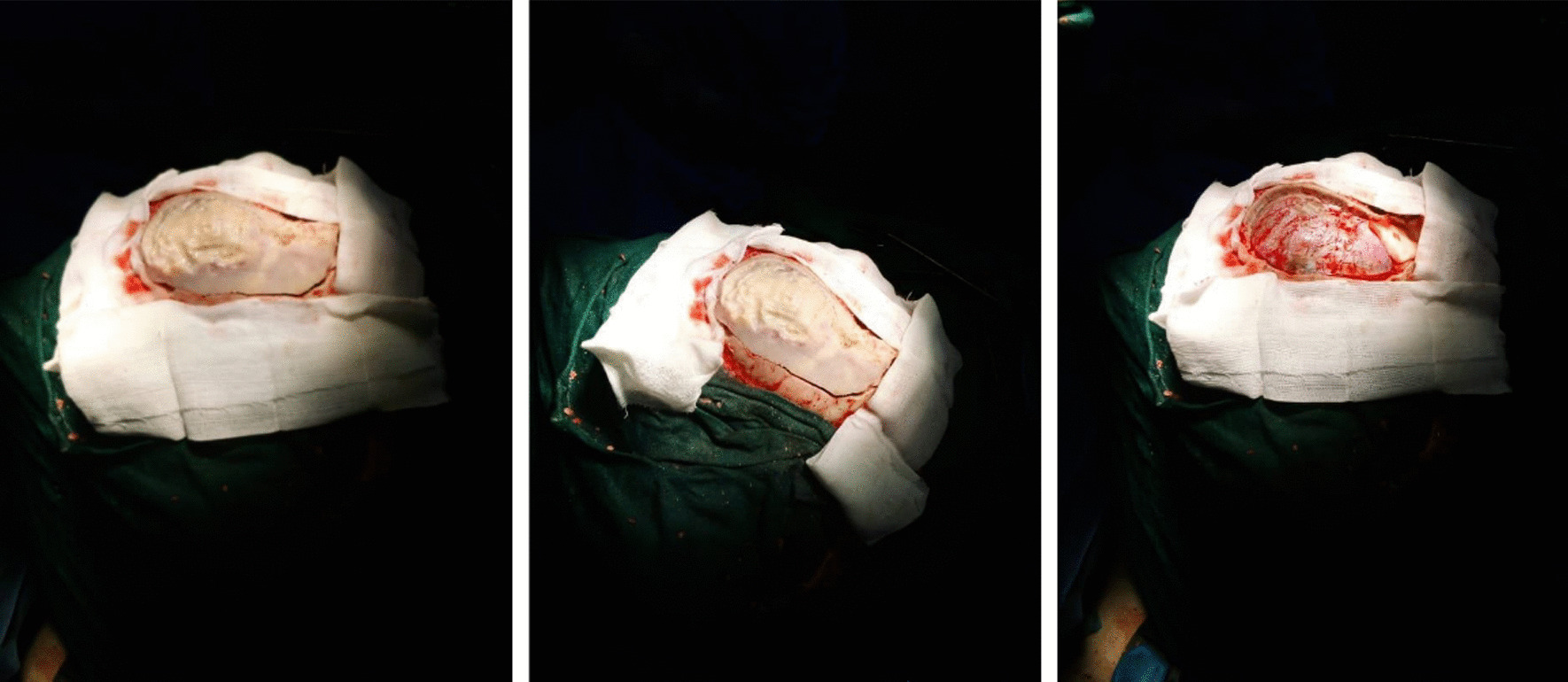
Tumor resection through craniotomy

**Fig. 4:**
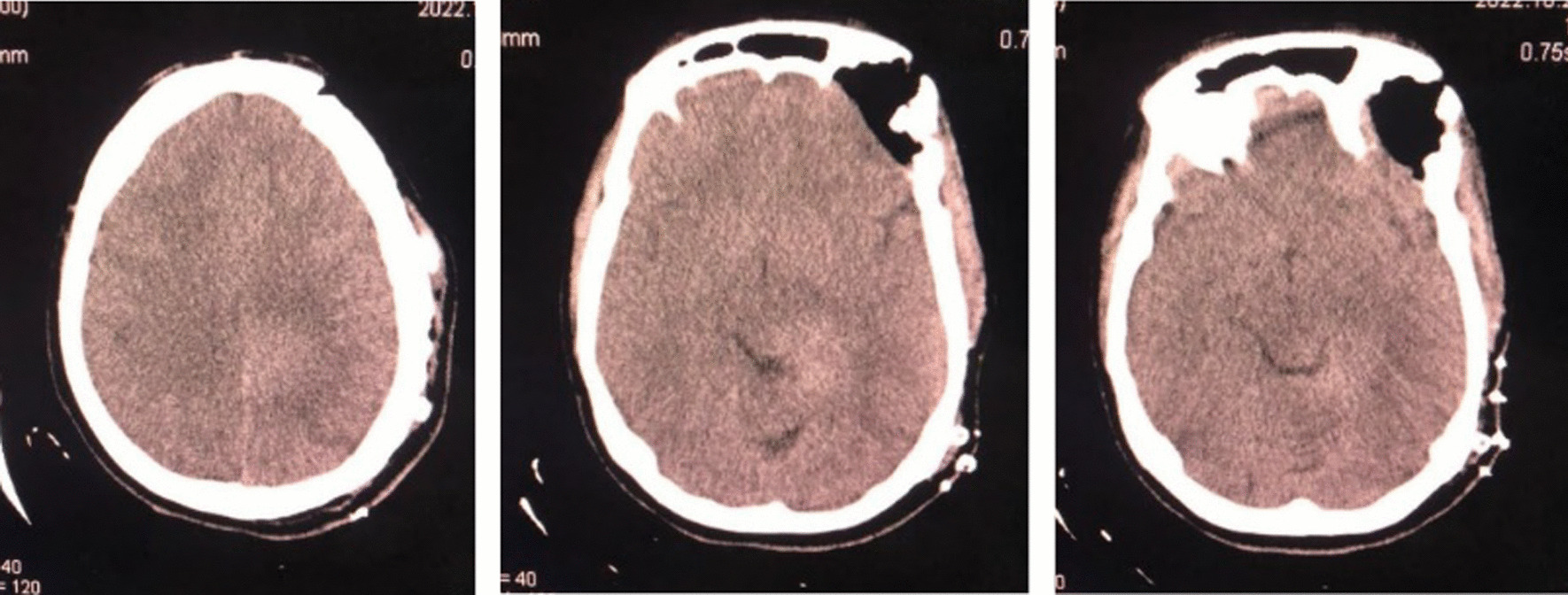
CT scan after surgery reveled normal findings with no residues

A 24-year-old Syrian woman came to our hospital complaining of headaches, syncope episodes, and blurred vision that started a month ago. The patient was conscious and reactive. The familial, allergic, and psychosocial histories of the patient were unremarkable. Medical and surgical histories revealed uncomplicated appendectomy and head trauma when she was 6 years old in a traffic accident. The patient did not undergo any investigations or treatment after the trauma. The physical examination was normal except for a huge-size tumor in the frontoparietal region of the head. All Laboratory tests were normal. Head computed tomography (CT) scan showed thickness in the space between the two bone plates in the left frontoparietal region, which reached the orbital roof without cortical destruction or periosteum reaction; the tumor size was 5 cm × 5 cm (Fig. 1). Magnetic resonance imaging (MRI) showed a heterogeneous bony tumor in the left frontoparietal region; the brain was within normal findings (Fig. 2). According to clinical examination, the patient’s history, and the CT scan findings, osteoma was suggested as a differential diagnosis. The surgical excision was indicated and medical consultations were performed before surgery were taken. Under general anesthesia, using the classic frontoparietal approach, the bone flap was removed. Then, the bony tumor was excised by using a high-speed pneumatic cranial drill (craniotome) avoiding dura matter laceration and brain damage (Fig. 3). After the surgery, one unit of blood was transfused because of mild bleeding during the surgery (patient’s hemoglobin was 9 mg/dl), and she was discharged after 2 days. The histopathology examination of the taken sample emphasized the diagnosis of osteoma. The follow-up for 7 months using laboratory and radiological investigations was uneventful, with disappearance of all the symptoms that were present, and aesthetically, the head shape returned to normal (Fig. 4).

## Discussion

Osteoma is a slow-growing benign tumor, commonly found in the skull bones [5]. The incidence rate for osteoma is 0.43% in the population [8]. It is seen between the fourth and sixth decades of life, with a prevalence in men [8]. This prevalence is explained by the larger size of the sinuses in men, and they are exposed to injuries more than women [8]. It is usually less than 10 mm, but in rare cases, it may reach > 30 mm (giant osteoma) [2, 4]. The growth rate of osteoma is very slow (1.61 mm/year); it takes between 12 and 30 years, and after Partial resection, relapse may still occur during 2–8 years [6]. There are three theories explaining the developing of osteoma. The first one suggests inflammation; chronic inflammation in the paranasal sinuses activates the formation of osteogenic cells related to the periosteum. The second theory encourages a congenital cause of the osteoma, especially when it arises in the bones of the vault. The last one indicates that trauma and muscle traction cause this tumor, as 30% of patients are found to have a history of head trauma [4, 6, 7]. Traumas could result in bleeding or swelling beneath the periosteum, and muscle tension could lift the periosteum in that area, triggering an osteogenic reaction. The initial trauma may be minor and easily overlooked by the patient, but ongoing muscle tension could sustain the response, leading to tumor growth [7, 9]. When the osteoma is multiple, it may be a part of Gardner’s syndrome, which also includes intestinal polyps, cysts, skin fibromas, and the presence of extra permanent teeth [1, 3]. Osteoma is often asymptomatic and diagnosed accidentally, but in some cases, it may cause mass-effect symptoms depending on the compressed organ next to it [1]. Headache is the most common symptom of intracranial osteoma [10]. Other symptoms include cerebrospinal fluid rhinorrhea, proptosis, diplopia, pneumatocele, brain abscess, and meningitis [2]. A CT scan is the cornerstone in the diagnosis and follow-up of osteoma and is essential in planning for resection, while the importance of MRI is in determining the relationship between the tumor and adjacent structures [7]. Osteoma appears in CT scans as a well-defined, round or oval, homogeneous, sessile, or pedunculated lesion [1, 5, 11], while it appears on MRI as a low-signal intensity lesion in all sequences [2]. According to radiological investigations, there are many differential diagnoses, such as exostosis, chronic focal sclerosing osteomyelitis, fibrous dysplasia, ossifying fibroma, chondroma, osteoblastoma, osteoid osteoma, osteosarcoma, odontoma, and Paget’s disease [2, 3, 5]. The final diagnosis of osteoma is made by histopathological examination, which divides the osteoma into two types: (1) compact osteoma (ivory), which is composed of mature lamellar bone and haversian canals without any fibrous tissues and (2) trabecular osteoma (mature), which is composed of cancellous trabecular bone, cortical bone margin, and bone marrow [1, 5]. Asymptomatic osteoma requires conservative treatment and continuous follow-up by CT [4]. Surgical treatment is required if it is large, causing a morphological deformity and pressuring nearby structures causing functional disorder [5].

## Conclusion

This paper highlights the importance of focusing on the medical history of patients with osteoma in an attempt to explain the reasons for its occurrence. It stresses the need to put osteoma within the differential diagnoses of the tumors that arise from the skull and encourages the development of investigations and therapeutic methods for the diagnosis and treatment of osteoma if it is located in rare places.

## Data Availability

Not applicable.

## Abbreviations

Head CT: Head computed tomography
MRI: Magnetic resonance imaging
